# The Failure of Thymol to Expel Whipworms (Trichuris Depressiuscula) from Dogs

**Published:** 1902-12

**Authors:** Ch. Wardell Stiles, Charles A. Pfender

**Affiliations:** Zoölogist of Bureau of Animal Industry; Student Assistant


### THE JOURNAL

OF

### COMPARATIVE MEDICINE AND VETERINARY ARCHIVES.

VOL. XXIII. *DECEMBER, 1902.* No. 12.

### THE FAILURE OF THYMOL TO EXPEL WHIPWORMS (TRICHURIS DEPRESSIUSCULA) FROM DOGS.

BY CH. WARDELL STILES, PH.D.,[1]
ZOÖLOGIST OF BUREAU OF ANIMAL INDUSTRY.,

AND

CHARLES A. PFENDER,
STUDENT ASSISTANT.

IN a series of experiments in the Zoological Laboratory of the Bureau of Animal Industry, doses of thymol varying from 10 to 80 grains (0.648 to 5.18 grammes) each were administered to eighteen dogs weighing from 8 to 35 pounds each, in order to test its efficacy in expelling whipworms (*Trichuris depressiuscula*). From the poor results obtained it must be concluded that this drug is very unsatisfactory in treating whipworm infections; in fact, that in safe doses it is practically useless. In several cases maw-worms (*Ascaris canis*) were passed, but in no case were all the specimens expelled. The drug had no effect on the double-pored tapeworms (*Dipylidium caninum*).

The best results were obtained by administering the drug in powdered form with castor oil. Thymol tablets were unsatisfactory, and thymol dissolved in alcohol, as advised by Theobald, is likely to be followed by serious convulsions, even by death; hence alcoholic solutions of the drug should be avoided. Theobald reports a case of partial prostration in a bull terrier from a dose of 3 grains (0.194 gramme); unfavorable symptoms developed in our dogs after 30 grains (1.94 grammes) in alcohol. In one case a dose of 20 grains (1.296 grammes) of thymol, administered in

[1] Transferred August 16, 1902, to Public Health and Marine Hospital Service as Chief of Division of Zoölogy, Hygienic Laboratory.

milk to a dog weighing 12 pounds, was followed by attempts to vomit. In the other cases, however, doses of 10 to 30 grains (0.648 to 1.94 grammes) of powdered thymol were administered to dogs weighing 8 to 15 pounds, and a dose of 75 grains (4.86 grammes) of powdered thymol was administered to a dog weighing 35 pounds without producing any unfavorable symptoms. Theobald's statement, therefore, that the dose for roundworms in dogs should not exceed 2 grains (0.129 gramme) does not apply to *powdered* thymol. Doses of 50 and 100 grains (3.24 to 6.48 grammes) of thymol in tablet form, and not followed by castor oil, administered to two dogs weighing 12 and 15 pounds, respectively, expelled all the whipworms (*Trichuris*), but made one dog slightly sick and the other dog very sick.

Thymol is one of the drugs which is most highly and most frequently recommended in uncinariasis (ankylostomiasis), and occasionally it is advised in cases of infection with whipworms (*Trichuris*), with maw-worms (*Ascaris*), with pinworms (*Oxyuris*), and with palisade-worms (*Strongylus*). Its use in human medicine in uncinariasis is common, and some authors warn against giving alcohol to a patient during the time that thymol is in his intestine. Theobald especially has recommended its use in horses, claiming that it acts not only upon the worms which are free in the horse's intestine, but also upon the worms in the mucosa. He directly advises the use of an alcoholic solution of the drug. Bozzolo (1881–82) and Sandwith (1894) advise that brandy be given after thymol in human practice.

Stiles (1901) used it in Texas in tablet form, powdered form, and alcoholic solution, experimenting with cattle, sheep, and goats.

The doses recommended by various authors for different animals may be seen from the following:

Man (*Homo sapiens*): It is usually advised that as an anthelmintic two doses of 2 grammes (31 grains) each be given two hours apart.

Bozzolo (1881-82) used as high as 10 grammes (154.32 grains) in a single day, giving the drug in five doses of 2 grammes (31 grains) each, and following the last dose with brandy.

Britto (1885) gave 6 grammes (92.6 grains) in a single day, administering the drug in three doses of 2 grammes (31 grains) each.

Graziadei (1882) used 12 grammes (185.18 grains) in a single day, administering six doses of 2 grammes (31 grains) each at intervals of two hours.

Campi (1886) used 8 grammes (123.45 grains) in a single day, giving the drug in twelve doses. He first gave 20 c.c. (5.41 drachms) of castor oil, then administered the drug every fifteen minutes. He followed the last dose with another administration of 20 c.c. (5.41 drachms) of castor oil. He also advises the use of rum or cognac during the treatment.[1]

Horse (*Equus caballus*): Theobald (1900, pp. 65, 66) administered 15 grains (0.972 gramme) to adults and 10 grains (0.648 gramme) to foals. He states that as much as 3 drachms (11.66 grammes) have been given to horses without serious effect.

Cattle (*Bos taurus*): Stiles (1901, p. 372) has given 240 grains (15.54 grammes) to a cow.

Sheep (*Ovis aries*) and goats (*Capra hircus*): Stiles (1901, p. 372) gave 32 to 60 grains (2.10 to 3.88 grammes), and later as high as 80 to 100 grains (5.18 to 6.48 grammes).

Dogs (*Canis familiaris*): Theobald (1898, p. 24) advises 2 grains (0.130 gramme) at the most, and states that 3 grains (0.194 gramme) have caused prostration. For our doses, see below.

Cats (*Felis domestica*): Theobald (1898, p. 24) advises 2 grains (0.130 gramme).

Fowls (*Gallus domesticus*): Theobald (1898, p. 24) advises 1 grain (0.065 gramme) for the expulsion of roundworms.

As stated above, we were recently led to try thymol on some dogs, in order to test its value when administered for the expulsion of whipworms (*Trichuris*); and although only eighteen experiments have thus far been made, it is believed that a short account of the results will be justified, and also that it will be of interest in connection with the subject of uncinariasis.

The dogs were obtained from the pound. Several days after being treated with thymol they were killed and examined for intestinal parasites, in order to see the efficacy of the doses adminis-

[1] Captain Fearnside, I. M. S., at the meeting of the British Medical Association at Ipswich, in 1900, gave a statistical table showing that he found the ova of *Uncinaria duodenalis* in convicts treated with thymol (20 grains [1.296 grammes] daily) over periods extending from ten to thirty days, just as numerous at the end of the treatment as at the beginning. It will be noted that the daily doses were only 20 or 30 grains [1.296 to 1.94 grammes]; also that the longest period of treatment was thirty days. Those who have had a large experience with this disease will not, therefore, accept such a table as proof either of the failure of thymol to effect a cure or as in any way upsetting the diagnosis of ankylostomiasis [uncinariasis].

On the other hand, most text-books appear to imply that it is only necessary to give a few (three or four) hourly doses of 20 to 30 grains of thymol, and possibly to repeat this treatment in another week or so, to effect a complete cure.

Ozzard says that he is prepared to state that in any case of ankylostomiasis [uncinariasis] ova of the *Uncinaria duodenalis* will always be found to be present after the first course, and in many cases even after the second or third courses of such treatment (Ozzard, A.T., 1902).

tered. The animals weighed from 8 to 35 pounds and ranged from about one to ten years old.

Attempts to give the drug in the regular food, such as milk and meat, were unsuccessful, since the dogs discovered even as low an amount as ½ grain (0.0324 gramme) in a saucer of milk, and refused to drink it. Administration was then practised as follows:

(*a*) In tablet form (10 grains each), thrown back on the tongue.

(*b*) In tablet form, together with castor oil.

(*c*) In powdered form in milk, administered by tube.

(*d*) In alcoholic solution, diluted with milk or water, administered by tube.

These experiments were made during the winter months (November, December, January) of 1901–1902 (excepting Dogs Nos. 17 and 18, which were treated in June, 1902), and the following parasitic worms were found post-mortem in the dogs examined:

| Name of parasite. | Present in. | Absent from. |
|---|---|---|
| Double-pored tapeworm (*Dipylidium caninum*) . . | 6 dogs. | 12 dogs. |
| Maw-worms (*Ascaris canis*) . . . . . . . . | 3 " | 15 " |
| Whipworms (*Trichuris depressiuscula*) . . . . | 15 " | 3 " |
| Hookworms (*Uncinaria canina*) . . . . . . . | 1 " | 17 " |

The number of dogs examined is too small to permit of any extensive generalization; but it is rather striking that after the treatment 83.33 per cent. showed infection with whipworms, 33.33 per cent. contained double-pored tapeworms, while 16.6 per cent. contained maw-worms, and only one case of infection with the hookworms was found. (This was in Dog No. 17, examined in June, 1902.) Considering the fact that hookworms are usually so common in dogs in the District of Columbia in the summer, we were hardly prepared not to find a single case of infection in the sixteen dogs examined in winter.

The experiments in detail were as follows:

Dog No. 1. Weight, 15 pounds. Received ten tablets, 10 grains each, of thymol; total, 100 grains (6.48 grammes). About four hours after receiving the drug the dog's temperature fell from 100° F. to 97° F., this low temperature continuing for about ten hours. Retching and vomiting set in, lasting about one hour. None of the tablets was found in the vomit. During this period the dog was apparently not suffering any serious pain. The fecal discharge the next day was normal, and contained several live whipworms (*Trichuris depressiuscula*). From now on the dog refused food, and had no further discharge from the bowels. Symptoms developed which indicated congestion of the intestine, and the animal died about eighty-four hours after dosing, just as we had determined to kill him.

Autopsy showed a highly congested condition of the stomach and small intestine. No worms were found.

Dog No. 2. Weight, about 12 pounds. Received 50 grains (3.24 grammes) of thymol in tablet form. Six hours after dosing the dog made several attempts to vomit, but there were no other signs of pain. The feces on the following morning contained a few live whipworms (*Trichuris depressiuscula*). The dog showed no signs of pain, but was killed about forty-eight hours after dosing. No lesions were found which could be attributed to the drug, while the cæcum contained large numbers of live whipworms (*Trichuris depressiuscula*).

Dog No. 3. Weight, about 12 pounds. Received 10 grains (0.648 gramme) of thymol in tablet form and 2 drachms (7.77 c.c.) of castor oil. No effect. Forty-eight hours later received 30 grains (1.94 grammes) of thymol in tablet form and 2 drachms (7.77 c.c.) of castor oil. Attempts to vomit about three hours later. No worms in the feces after this dose. Killed several days later, and a number of live whipworms (*Trichuris depressiuscula*) were found in the cæcum.

Dog No. 4. Weight, not over 15 pounds. Received 10 grains (0.648 gramme) of thymol in tablet form and 2 drachms (7.77 c.c.) of castor oil. No effect. Forty-eight hours later received 20 grains (1.296 grammes) of thymol in tablet form and 4 drachms (15.55 c.c.) of castor oil. No worms were passed. Killed twenty-four hours later, and cæcum found heavily infected with live whipworms (*Trichuris depressiuscula*).

Dog No. 5. Weight, not over 15 pounds. Received 20 grains (1.296 grammes) of thymol in tablet form and 4 drachms (15.55 c.c.) of castor oil. No worms discharged. Killed forty-eight hours after dosing, and a number of live whipworms (*Trichuris depressiuscula*) were found in the cæcum.

Dog No. 6. Same as No. 5. Same result.

Dog No. 7. Weight, 12 pounds. Received 20 grains (1.296 grammes) of powdered thymol in milk. Shortly afterward frequent attempts to vomit. Forty-eight hours later same size dose repeated. No worms in the droppings. Forty-eight hours later same size dose repeated. No worms in the droppings. Dog was killed about one hundred and twenty hours after the first dose, and several live whipworms were found loose in the intestine.

Dog No. 8. Weight, not over 15 pounds. Received 20 grains (1.296 grammes) of powdered thymol in milk. Forty-eight hours later same size dose repeated; again, forty-eight hours later, same size dose, with 4 drachms (15.55 c.c) of castor oil. Shortly after the third dose slight convulsions were noticed, lasting several hours and followed by great depression. At no time during the treatment were worms found in the droppings. Forty-eight hours after the third dose the animal was killed. Numerous live whipworms (*Trichuris depressiuscula*) were found in the cæcum.

Dog No. 9. Weight, not over 15 pounds. Received 20 grains (1.296 gramme) of powdered thymol. Forty-eight hours later the dose was repeated, but no worms were expelled. After another forty-eight hours the dose was again repeated, with the addition of 2 drachms (7.77 c.c.) of castor oil; still no worms were expelled. The animal was killed twenty-four hours later, and the cæcum was found very heavily infected with live whipworms (*Trichuris depressiuscula*).

Dog No. 10. Weight, not over 15 pounds. Received 10 grains (0.648 gramme) of powdered thymol. Killed three days later. Live whipworms (*Trichuris depressiuscula*) were found in the cæcum, and several live specimens of the double-pored tapeworm (*Dipylidium caninum*) were found in the small intestine.

Dog No. 11. Same data as Dog No. 10, except that no tapeworms were present.

Dog No. 12. Weight, 15 pounds. Received 10 grains (0.648 gramme) of powdered thymol and 2 drachms (7.77 c.c.) of castor oil. Two maw-worms (*Ascaris canis*) were expelled several hours after the administration of the drugs. The dog was killed twenty-four hours later, and several live maw-worms (*Ascaris canis*) and tapeworms (*Dipylidium caninum*) were present.

Dog No. 13. Weight, not over 15 pounds. Received 10 grains (0.648 gramme) of powdered thymol and 2 drachms (7.77 c.c.) of castor oil. The fecal discharge six hours later contained several maw-worms (*Ascaris canis*). Four days later 20 grains (1.296 gramme) of powdered thymol were given, but no worms were passed. Ten days after the second dose the dog received 30 grains (1.94 grammes) of thymol in alcohol. About an hour later convulsions set in, lasting for six hours. No worms were expelled. The next day the dog was killed, and several live maw-worms (*Ascaris canis*), together with quite a heavy infection of live tapeworms (*Dipylidium caninum*), were found.

Dog No. 14. Weight, 14 pounds. Received 30 grains (1.94 grammes) of thymol in alcohol. Intense convulsions developed within a few minutes and lasted several hours. No worms were passed. The dog was killed four days later, and the cæcum was found infested with live whipworms (*Trichuris depressiuscula*), and the small intestine contained several tapeworms (*Dipylidium caninum*).

Dog No. 15. Weight, 35 pounds. Received 75 grains (4.86 grammes) of thymol in tablet form. No inconvenience whatever was observed; no worms were discharged. Forty-eight hours later the dog received 75 grains (4.86 grammes) of thymol dissolved in alcohol; no results. Ninety-six hours after the second dose the dog received 80 grains (5.18 grammes) of thymol in powdered form ; no results. Upon killing this dog two days later several specimens of live maw-worms (*Ascaris canis*) were found. There was a slight congestion of the abdominal cavity, but otherwise nothing abnormal was noticed, despite the large doses which had been given.

Dog No. 16. Weight, 8 pounds. Received 30 grains (1.94 grammes) of thymol in tablet form. No results. Two days later 30 grains (1.94 grammes) of thymol were administered in alcohol. No results. Four days later 40 grains (2.59 grammes) of powdered thymol were administered. No results. The dog was killed the next day, and several live whipworms (*Trichuris depressiuscula*) were found in the cæcum.

Dog No. 17. Weight, about 15 pounds. Received 20 grains (1.296 grammes) of thymol in tablet form daily for five days in succession. A microscopic fecal examination showed infection with whipworms (*Trichuris depressiuscula*) and hookworms (*Uncinaria canina*). The administration of thymol in daily doses of 20 grains (1.296 grammes) was continued after one day's intermission. On the seventh day following the microscopic examination of the feces there appeared a slight diarrhœa, which, however, soon subsided, leaving the animal in apparently good health. Two days later the daily doses were recommenced, and were continued for six days without producing any serious symptoms. The dog was killed after having received 20 grains (1.296 grammes) of thymol daily for sixteen days in succession, excepting the intermissions mentioned. Several live maw-worms (*Ascaris canis*), together with several hookworms (*Uncinaria canina*), were found in the intestine, while the cæcum showed quite a heavy infection of whipworms (*Trichuris depressiuscula*).

Dog No. 18. Weight, 17 pounds. Received 20 grains (1.296 grammes) of thymol in tablet form for twenty consecutive days, Sundays excepted. Fecal examination at various times showed an occasional discharge of maw-worms (*Ascaris canis*). On the sixteenth day of the treatment the dog became very sick, being taken with violent fits of trembling, and vomiting the medicine which had been administered a few hours prior to his first attack. He recovered the following day, and at no time afterward presented any marked indisposition.

He was killed twenty days after receiving the last dose. The walls of the cæcum were found literally covered with whipworms (*Trichuris depressiuscula*), and several tapeworms (*Dipylidium caninum*) were present in the intestine.

